# 基于液相色谱-质谱代谢组学方法研究中药定喘汤对呼吸道合胞病毒感染的疗效

**DOI:** 10.3724/SP.J.1123.2020.06013

**Published:** 2021-03-08

**Authors:** Yang OUYANG, Lei CHI, Chao XU, Xinjie ZHAO, Zhenze CUI

**Affiliations:** 1.中国科学院分离分析化学重点实验室, 中国科学院大连化学物理研究所, 辽宁 大连 116023; 1. Chinese Academy of Sciences Key Laboratory of Separation Science for Analytical Chemistry, Dalian Institute of Chemical Physics, Chinese Academy of Sciences, Dalian 116023, China; 2.中国科学院大学, 北京 100049; 2. University of Chinese Academy of Sciences, Beijing 100049, China; 3.大连医科大学附属大连市儿童医院, 辽宁 大连 116012; 3. Dalian Children’ s Hospital of Dalian Medical University, Dalian 116012, China;

**Keywords:** 液相色谱-串联质谱法, 呼吸道合胞病毒, 代谢组学, 中药, 定喘汤, liquid chromatography-tandem mass spectrometry (LC-MS/MS), respiratory syncytial virus (RSV), metabolomics, Chinese medicine, asthma-relieving decoction

## Abstract

呼吸道合胞病毒(RSV)容易引发下呼吸道感染,尤其是小儿毛细支气管炎。中药定喘汤用于RSV感染的治疗在中国有着广泛的临床实践基础。由于中药体系的复杂性,其治疗机制和主要药效成分还不够明确。采用代谢组学方法分析中药药效,可以为传统中药治疗提供现代科学论证。该研究对大鼠各干预组分别采用中药定喘汤全方,宣、降、清分解剂以及利巴韦林灌胃治疗,正常组大鼠和RSV模型组大鼠均采用生理盐水灌胃。分别取大鼠在第0、3、7天的眼底血浆进行基于液相色谱-串联质谱法的非靶向代谢组学分析,以研究RSV感染引起的代谢组改变、定喘汤对RSV感染相关代谢物的调控及定喘汤的主要药效来源。对检测的代谢物离子进行多变量分析,建立主成分分析模型,结果显示在RSV感染及治疗进程中,中药定喘汤组与利巴韦林阳性对照组对RSV感染大鼠产生的总体代谢调控效果类似。在3种定喘汤分解剂中,组成成分为麻黄和白果的宣法分解剂对代谢组的调控作用与定喘汤更为接近,可能为定喘汤的主要药效来源。基于配对*t*检验发现大鼠在感染RSV后有83种代谢物发生了显著性改变,表明RSV感染可造成大鼠多条代谢通路紊乱。第3天时中药定喘汤对包括胆汁酸、氨基酸、有机酸、脂质等在内的代谢物有治疗效果。3种分解剂中,宣法分解剂对代谢物的调节作用与定喘汤类似,而降法分解剂和清法分解剂对大鼠因感染RSV引起的酰基肉碱的异常升高有明显下调作用。各组都有维持肠道菌群和免疫系统稳定的药效。该研究采用代谢组学方法评价定喘汤的药效,发现了定喘汤治疗后改变的代谢物的种类及变化规律,为定喘汤的治疗机理和活性成分的研究提供了理论支持。

## Abstract (en)

Respiratory syncytial virus (RSV) can cause lower respiratory tract infections, such as bronchiolitis in infants. In China, traditional asthma-relieving medicine has numerous clinical applications in the treatment of RSV infections. However, due to the complexity of the traditional Chinese medicine system, its therapeutic mechanism and main pharmacological components remain unclear. Metabolomics can be used to analyze the efficacy of traditional Chinese medicine to provide modern scientific evidence for such treatments. In this study, an animal model experiment was performed with seven groups of three-week-old rats. The model group and five intervention groups were inoculated nasally with RSV for three consecutive days, and the normal group was treated with the same amount of saline for three consecutive days under the same conditions. In parallel, the five intervention groups were treated separately with the following via intragastric administration for seven consecutive days: asthma-relieving traditional Chinese medicine decoction, its three constituent agents (ascending (xuan) therapy, descending (jiang) therapy, pyretic clearing (qing) therapy), and ribavirin. Both normal group and RSV model group were administered with normal saline via intragastric administration as controls for seven consecutive days. The fundus plasma of rats in each group was collected on day 0, day 3, and day 7. Liquid chromatography-mass spectrometry-based untargeted metabolomics analysis was performed to investigate the changes in the metabolome after RSV infection, the effects of the asthma-relieving decoction on the regulation of metabolites related to RSV infection, and the primary source of efficacy. The detected metabolite ions were corrected using internal standards. Multivariate analysis of ions with an RSD value of less than 30% in quality control (QC) samples was used to construct principal component analysis models to monitor the overall metabolic changes of each group. The results showed that, during RSV infection and treatment, the asthma-relieving decoction and the positive control ribavirin had similar effects on the overall metabolic regulation of RSV-infected rats. Among the three asthma-relieving decoction constituent agents, the ascending (xuan) therapy agents which was composed of ephedra and ginkgo had a closer metabolic regulation effect with asthma-relieving decoction, and might be the main source of pharmacological efficacy. Based on the retention time, *m/z* value and tandem mass spectra in the database established by our laboratory, a total of 150 metabolites were identified. Paired *t*-tests were performed using data of the identified metabolites before and after RSV infection in each group, and it was found that 83 metabolite levels significantly changed after RSV infection, indicating that RSV infection could lead to disorders of multiple metabolic pathways in rats. The altered pathways included aminoacyl-tRNA biosynthesis, phenylalanine, tyrosine, and tryptophan biosynthesis, primary bile acid biosynthesis, phenylalanine metabolism and sphingomyelin metabolism. On the third day, the asthma-relieving decoction had regulatory effects on several metabolites such as bile acids, amino acids, organic acids, lipids, etc. Among the three asthma-relieving decoction constituent agents, the ascending (xuan) therapy agents had more similar effects on the regulation of metabolites with the asthma-relieving decoction. On the other hand, the descending (jiang) therapy agents and pyretic clearing (qing) therapy agents down-regulated the abnormal increase in acylcarnitine caused by the RSV infection. Additionally, both asthma-relieving decoction and its constituent agents could maintain the stability of the immune system and metabolism of the intestinal flora in rats. This study used metabolomics to evaluate the efficacy of an asthma-relieving decoction and demonstrate the metabolites and the corresponding changes after asthma-relieving decoction-based treatment. It provides theoretical support for research on the therapeutic mechanism and active ingredients of asthma-relieving decoction.

呼吸道合胞病毒(respiratory syncytial virus, RSV)是单股负链RNA病毒。它是引发毛细支气管炎等呼吸道感染的病原之一^[1]^。该病毒对婴幼儿的伤害尤其大^[2]^,感染后极易发展为呼吸衰竭和重度肺炎^[3]^,严重时会导致死亡。目前全球尚无统一有效的治疗方法或疫苗。西药利巴韦林是一种核苷类药物,可插入病毒的RNA基因链中导致基因错误突变,是一种广谱的抗病毒药^[4]^。已有研究证明利巴韦林对RSV感染有疗效^[5,6]^。它目前已被美国食品药品管理局批准以雾化形式用于治疗RSV感染^[7]^,但是利巴韦林也被报道易导致并发症^[8]^和耐药性突变^[9]^。传统中药用于RSV感染的治疗在中国有着广泛的临床实践基础。《摄生众妙方》记载了中药定喘汤的配方,该药物可用于治疗咳嗽喘息。定喘汤可通过抑制病毒复制减少病毒对机体的伤害^[10]^。同时它也能修复RSV感染造成的机体天然免疫系统和获得性免疫系统的紊乱,提高机体自身免疫力以抵抗病毒^[11]^。此外定喘汤也可以通过下调小鼠TLR4和NF-κB mRNA的表达,减少因RSV感染引起的炎症反应^[12]^。

生物体内代谢物的变化能及时反映出机体正在发生的变化,而代谢组学是研究代谢物变化规律的科学,能系统地阐述机体代谢组的动态改变^[13]^。目前代谢组学已经被用于研究RSV感染后细胞^[14]^、尿液^[15]^、血液和组织^[16]^中的代谢物改变。研究人员通过核磁共振代谢组学方法,发现RSV感染婴儿的尿液中有13种代谢物与健康对照相比发生了显著性变化,包括1-甲基烟酰胺、4-脱氧苏糖酸、肌酸、2-氨基丁酸和琥珀酸等^[15]^。基于液相色谱-串联质谱(LC-MS/MS)平台对RSV肺炎小鼠模型的脂质代谢组学研究表明,小鼠肺组织和血浆中分别有多种脂质,包括磷脂、鞘脂、脂肪酸等发生代谢异常^[16]^。代谢物组学也被用于评价药物对RSV感染的治疗效果以及药物治疗机理研究^[17,18]^。基于LC-MS的代谢组学分析发现中成药金欣口服液可以改善RSV感染后血浆和肝组织中氨基酸代谢以及脂质代谢的紊乱^[17]^。基于气相色谱-串联质谱平台的研究发现中药黄芩中天然活性成分黄芩素能改善RSV感染小鼠的肝脏组织中天冬氨酸、甘氨酸和磷酸肌酐等相关代谢通路的失衡^[18]^。我们之前的研究也发现了定喘汤能有效改善RSV感染后多种胆汁酸异常,进而保护肝肠循环,维持肠道代谢稳定^[19]^。

本研究采用基于LC-MS的非靶向代谢组学方法^[20]^,研究大鼠感染RSV后血浆代谢的改变及中药定喘汤对RSV感染大鼠的血液代谢异常的治疗效果,并通过研究定喘汤的3种分解剂,考察定喘汤的主要药效来源,以期能为RSV的药物治疗研究提供依据。

## 1 实验部分

### 1.1 仪器与试剂

1.1.1 大鼠、病毒和药品信息

SPF级Wistar幼龄大鼠(3周,辽宁本溪长生生物技术有限公司),呼吸道合胞病毒(RSV,中国预防医学科学院病毒所毒种室),中药组成和制备过程与我们之前发表的工作^[21]^一致。西药利巴韦林来自四川百利药业,用生理盐水制成10 mg/mL备用。所有中药材均来自辽宁中医药大学附属医院。定喘汤组由麻黄9 g、杏仁9 g、半夏9 g、苏子6 g、黄芩12 g、桑白皮9 g、白果9 g、款冬花9 g、甘草3 g组成;宣法分解剂由麻黄9 g、白果9 g组成;降法分解剂由杏仁9 g、半夏9 g、苏子6 g、款冬花9 g组成;清法分解剂由黄芩12 g、桑白皮9 g组成。将以上药物按照传统方法水煎2次混合后,制成含生药2 g/mL的药液备用。

1.1.2 试剂

流动相和内标均为UPLC级别。其中,乙腈和甲醇来自Merck公司(达姆施塔特,德国),甲酸和内标均来自Sigma-Aldrich公司(密苏里州圣路易斯,美国)。将内标配制于甲醇中用于血浆代谢物的提取。内标的种类及含量如下:色氨酸-d_5_(L-tryptophan-d_5_, 4.26 μg/mL)、苯丙氨酸-d_5_(L-phenyl-d5-alanine, 3.61 μg/mL)、胆酸-d_4_(cholic acid-d_4_, 1.85 μg/mL)、鹅脱氧胆酸-d_4_(chenodeoxycholic acid -d_4_, 1.48 μg/mL)、棕榈酸-d_3_(palmitic acid-d_3_, 2.50 μg/mL)、硬脂酸-d_3_(stearic acid-d_3_, 2.50 μg/mL)、肉碱C2∶0-d_3_(carnitine C2∶0-d_3_, 0.16 μg/mL)、肉碱C10∶0(carnitine C10∶0-d_3_, 0.10 μg/mL)、肉碱C16∶0-d_3_(carnitine C16∶0-d_3_, 0.15 μg/mL)、胆碱-d_4_(choline-d_4_, 0.26 μg/mL)、溶血磷脂酰胆碱19∶0(lysophosphatidylcholine 19∶0, LPC 19∶0, 0.75 μg/mL)、磷脂酰胆碱38∶0(phosphatidylcholine 38∶0, PC 38∶0, 0.63 μg/mL)和鞘磷脂12∶0(sphingomyelin 12∶0, SM 12∶0, 0.13 μg/mL)。

1.1.3 主要设备

全外排二级生物安全柜(SG403TXCE, Baker公司,桑福德,美国); 96孔除蛋白板(Phenomenex公司,托兰斯,美国); 96孔板正压装置(Waters公司,米尔福德,美国); Waters ACQUITY超高效液相色谱(Waters公司,米尔福德,美国)串联TripleTOF 5600质谱(AB SCIEX公司,弗雷明汉,美国)。

### 1.2 动物实验及样本收集

动物实验设计如图1所示。将42只Wistar大鼠随机平均分为7组,分别为正常组(简写Z)、模型组(简写M)、利巴韦林组(简写L)、定喘汤组(简写D)、宣法组(简写D-X)、降法组(简写D-J)和清法组(简写D-Q),每组6只。模型组和各干预组连续3天滴鼻接种RSV(0.1 mL/只)。正常组在相同条件下连续3天接种等量生理盐水。各干预组在第1次滴鼻2 h后开始灌胃给药干预,正常组和模型组在相同条件下给予生理盐水灌胃,各组持续灌胃干预7天。其中利巴韦林组给药量为每天0.1 mL/kg的利巴韦林药液,作为阳性对照。定喘汤组及其分解剂组每天分别给予2 g/kg中药药液。取各组第0天(接种RSV前的一天)、第3天和第7天的眼底血,加入低分子肝素抗凝,以转速3000 r/min离心5 min。取上层血浆保存在-80 ℃,用于后续的代谢组学分析。按照相同分组、感染和喂养方式处理另外两批老鼠,分别于第3天和第7天处死,提取肺组织采用实时荧光定量PCR方法^[22]^检测RSV mRNA表达(即病毒载量)。计算第7天处死大鼠的肺组织占体重的百分比,得到第7天的肺指数。

**图1:**
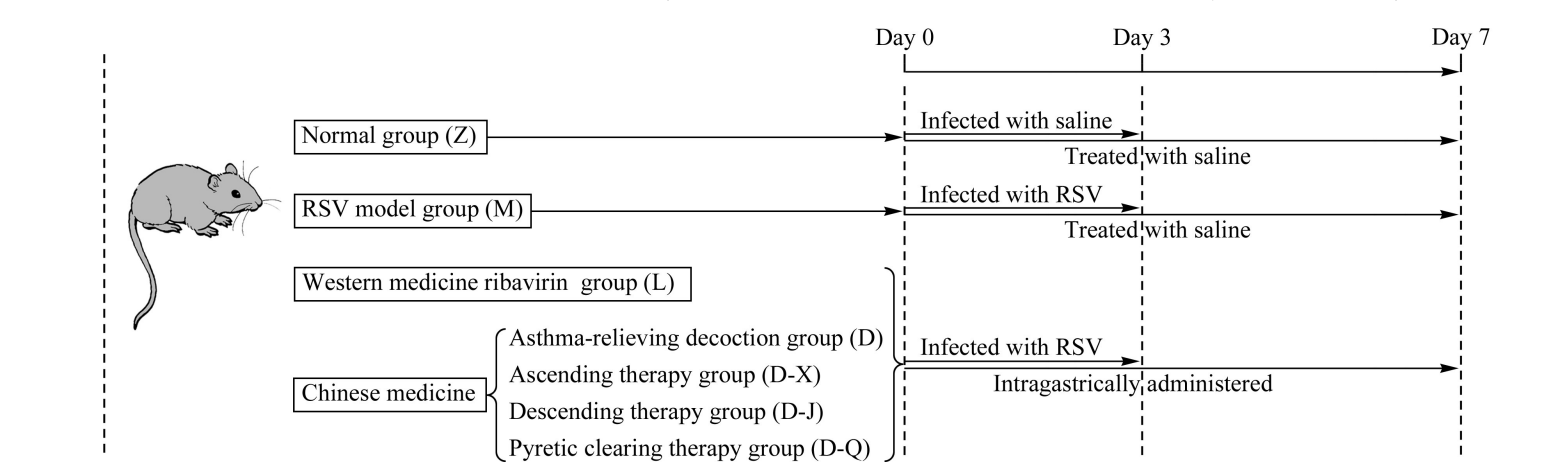
大鼠喂养、感染和给药过程图示

### 1.3 血浆样品预处理

大鼠血浆从-80 ℃取出后于冰水中融化,涡旋1 min。所有大鼠样品随机排序进行预处理与检测。处理方法如下:取50 μL血样与200 μL含内标的甲醇于96孔板孔内。室温下涡旋10 min混合均匀后静置10 min。用正压过滤的方式去除蛋白。将过滤后的提取溶液真空低温冻干,并保存在-80 ℃冰箱。每个样品用50 μL体积比为1∶4的甲醇-水溶液复溶后进行LC-MS分析。另将每个大鼠血浆取10 μL混合均匀,制备为质量控制(QC)样品。QC样品的处理同大鼠血浆样品。每检测10个大鼠血浆样品后检测一个QC样品,以评估检测过程的稳定性。

### 1.4 UPLC-MS条件

分析过程采用我们之前建立的液相色谱-质谱联用快速分析技术^[20]^,分析柱使用ACQUITY UPLC BEH C8色谱柱(50 mm×2.1 mm, 1.7 μm, Waters公司,米尔福德,美国)。色谱的流动相A相和B相分别为0.1%(v/v)的甲酸水溶液和0.1%(v/v)的甲酸乙腈溶液。流速为0.4 mL/min。柱温为60 ℃。进样体积为5 μL。初始梯度为95%的A相,平衡0.5 min。然后在1.5 min内线性变换至60%的A相。再之后在6 min内线性变换至0%的A相,并保持2 min。最后在0.1 min内线性变化为95%的A相,并平衡1.9 min。总的液相色谱分离时间为12 min。质谱的鞘气和气帘气压力分别为379和241 kPa。质谱扫描范围为*m/z* 80~1000, 信息依赖型采集(IDA)模式扫描范围为*m/z* 60~1000。正离子模式下,喷雾电压为5.5 kV,离子源温度为550 ℃。负离子模式下,喷雾电压为-4.5 kV,离子源温度为450 ℃。

### 1.5 数据处理方法

用MarkerView软件(1.2.1版本,AB SCIEX公司,康科德,加拿大)提取离子原始峰表。用80%规则^[23]^对离子峰表预处理,并采用内标进行校正。将在QC中RSD值小于30%的离子导入SIMCA-P软件(11.0版本,Umetrics AB公司,于默奥,瑞典)进行多变量分析,得到主成分分析(PCA)模型。将各组校正到正常组后,在IBM SPSS软件(25.0版本,IBM公司,阿蒙克,美国)中对根据实验室数据库^[24]^定性的150个代谢物做*t*检验。其中*p*值小于0.05的为有显著性差异。在MetaboAnalyst(https://www.metaboanalyst.ca/)网站进行通路分析。采用Multi Experiment Viewer软件(4.7.4版本)^[25]^绘制热图。

## 2 结果与讨论

### 2.1 临床指标评价

病毒载量和肺指数可监测疾病的进程,判断药物疗效。对病毒载量和肺指数数据进行独立样本*t*检验,考察各组大鼠与模型组之间的差异,结果见图2。如图2a、2b所示,模型组第3天和第7天的病毒载量分别为2.38±0.07、1.26±0.05,说明第3天RSV感染程度显著高于第7天;利巴韦林组和定喘汤组的病毒载量与相同时间点模型组比较都有显著性下降,且这两个用药组之间无显著差异;各中药分解剂组与模型组相比,清法组第3天和第7天的病毒载量显著性降低,宣法组的病毒载量在给药后的第7天也显著性减少。

**图2:**
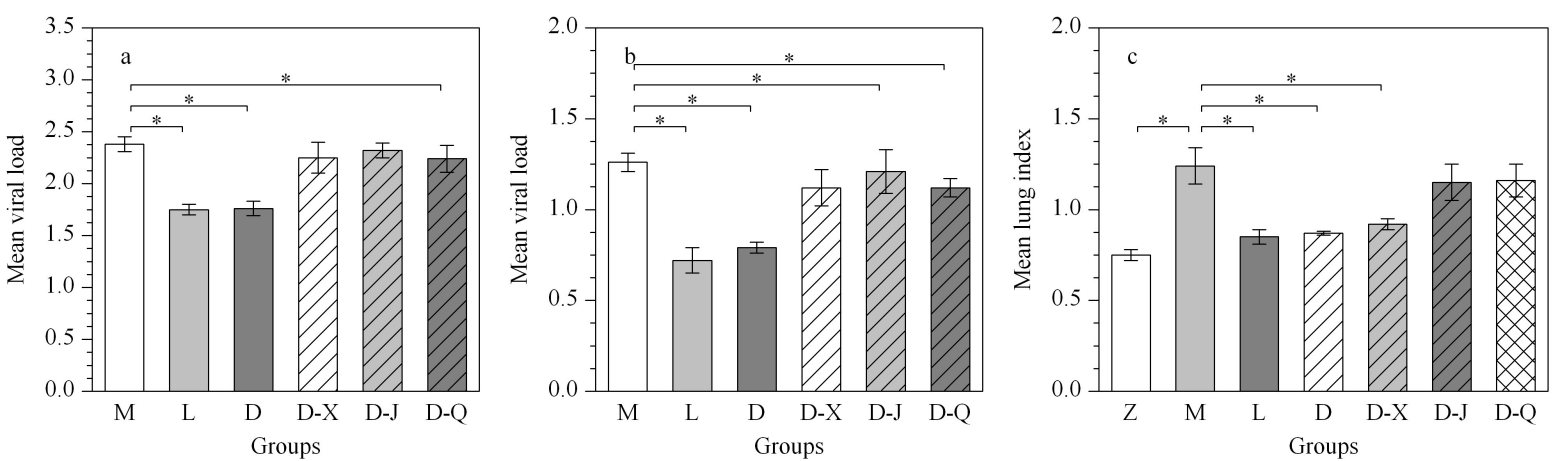
各组大鼠的平均病毒载量和平均肺指数柱状图

图2c表明模型组与正常组相比,第7天的肺指数显著增高。利巴韦林和定喘汤组与模型组相比,都显著降低了肺指数,且两个用药组之间并无显著差异,说明这两种药物都对RSV感染造成的肺指数增加有一定疗效。3种中药分解剂中仅宣法分解剂组显著性降低了肺指数。

### 2.2 代谢组学数据评价

本研究采用的是我们已建立的基于LC-MS技术的非靶向代谢组学方法,该方法能提高样品处理和分析通量,方法建立及考察过程参考文献^[20]^。基于非靶向代谢组学方法对正常组、模型组、利巴韦林组、定喘汤组、宣法组、降法组和清法组共计42只大鼠的血浆进行分析,在Waters ACQUITY超高效液相色谱-串联TripleTOF 5600质谱上采用正、负离子两种模式检测。正离子模式和负离子模式下分别检测到1835和1906个离子。QC样品中RSD值小于30%的离子累计响应分别占正、负离子模式总响应的84.9%和92.9%(如图3所示),该结果表明样品处理和检测过程稳定可靠。

**图3:**
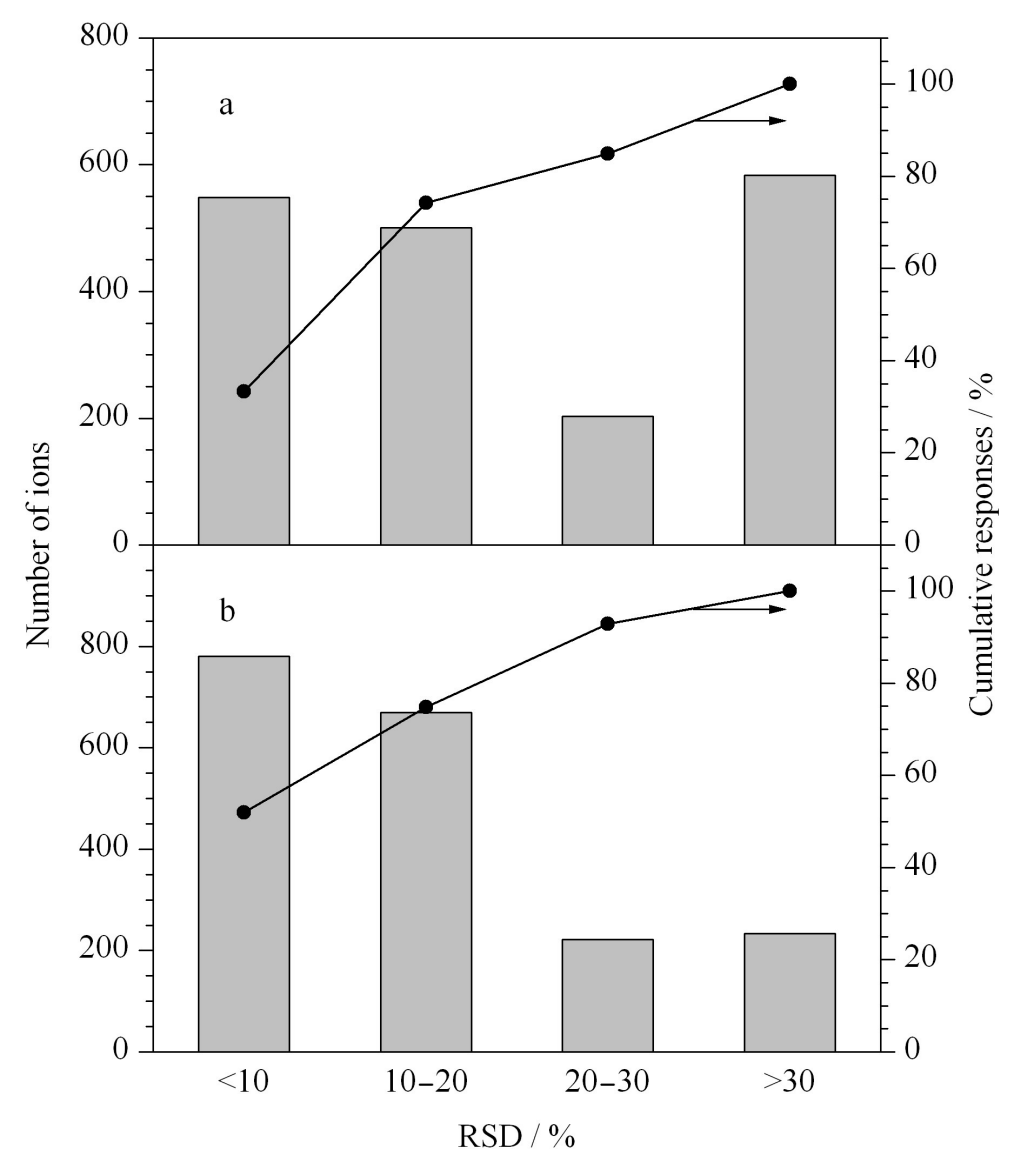
(a)正离子和(b)负离子检测模式下质量控制(QC)样品的RSD值

### 2.3 模式识别分析

为了从代谢物组学角度整体评价药物的药效,本研究对第0天、第3天和第7天样本数据分别建立PCA模型,得分图见图4。从图4a可以看到,第0天各组无明显分离趋势,说明造模前各组样本无差异。如图4b所示,第3天时正常组和模型组显示出明显分离趋势;模型组、清法组和降法组有聚合趋势;定喘汤组、宣法组和利巴韦林组有聚集趋势,且与模型组有明显的分离趋势,说明定喘汤、宣法分解剂与利巴韦林对RSV感染大鼠产生的代谢调控效果类似,而清法分解剂和降法分解剂对RSV感染大鼠的代谢调控不显著。通过图4b中PCA得分图显示的宣法组与定喘汤组聚集在同一区域,我们推测宣法分解剂在定喘汤的代谢调控功能中起主要作用,可能为定喘汤的主要药效来源。与第3天相比,第7天时各组与正常组间呈现明显聚拢趋势(如图4c所示),说明感染RSV第7天时,各组的代谢组趋于正常。

**图4:**
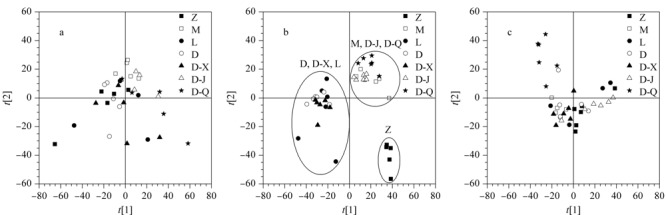
(a)第0天、(b)第3天和(c)第7天的主成分分析(PCA)得分图

### 2.4 关键代谢物筛选及变化规律

为了排除生长、取血等过程对大鼠代谢组的影响,将各组的代谢物峰强度数据校正到相对应天数的正常组数据。为了找出感染RSV引起的代谢改变,对模型组第3天和第0天的代谢组数据进行配对样本*t*检验,得到并定性出83个发生显著性变化的代谢物(*p* < 0.05)。将这83个与RSV感染密切相关的代谢物导入MetaboAnalyst网站进行通路分析,发现多条代谢通路在RSV感染后的第3天存在明显改变,改变的通路有氨酰基-tRNA生物合成,苯丙氨酸的代谢,苯丙氨酸、酪氨酸和色氨酸的生物合成,初级胆汁酸的生物合成,鞘磷脂代谢等(见图5)。

**图5:**
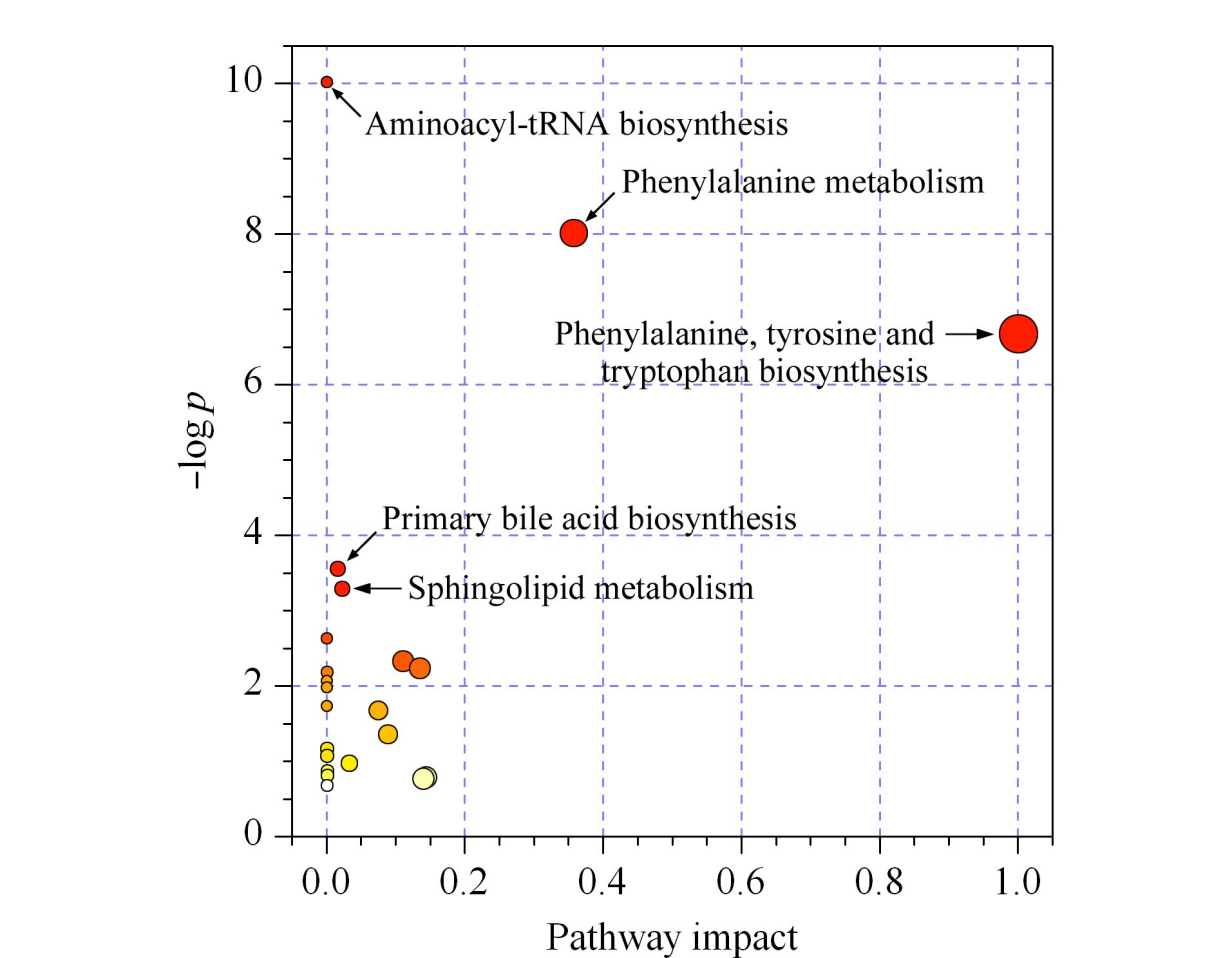
RSV感染对大鼠代谢通路的影响

接下来我们重点关注各药物组对这83个因RSV感染而显著性改变代谢物的调节。分别对各组第3天和第7天与自身第0天的代谢组数据做配对*t*检验。第3天时,由于接种病毒的同时给予药物干预,与RSV感染引起的变化趋势相同且有显著性差异(*p*< 0.05)的代谢物则认为药物干预对该代谢物无显著性药效,否则即药物对该代谢物产生了药效。与RSV感染变化趋势相同且有显著性差异的代谢物个数总结于图6a。如图6a所示,第3天时各干预组中与RSV感染有相同变化趋势的代谢物的个数相对于模型组都大大减少,说明各给药组均可对代谢物产生药效。第7天时(如图6b所示),由于已经停止接种病毒,模型组中与RSV感染有相同变化趋势的代谢物个数减少,说明感染RSV后,即使未给予药物治疗,机体也能产生一定的自愈能力,这符合病毒感染的临床实际。而定喘汤组和利巴韦林组中有显著性差异的代谢物明显少于模型组和3个中药分解剂组,说明定喘汤组和利巴韦林组在感染第7天时对与RSV感染密切相关的代谢物产生药效,且药效优于各中药分解剂组。

**图6:**
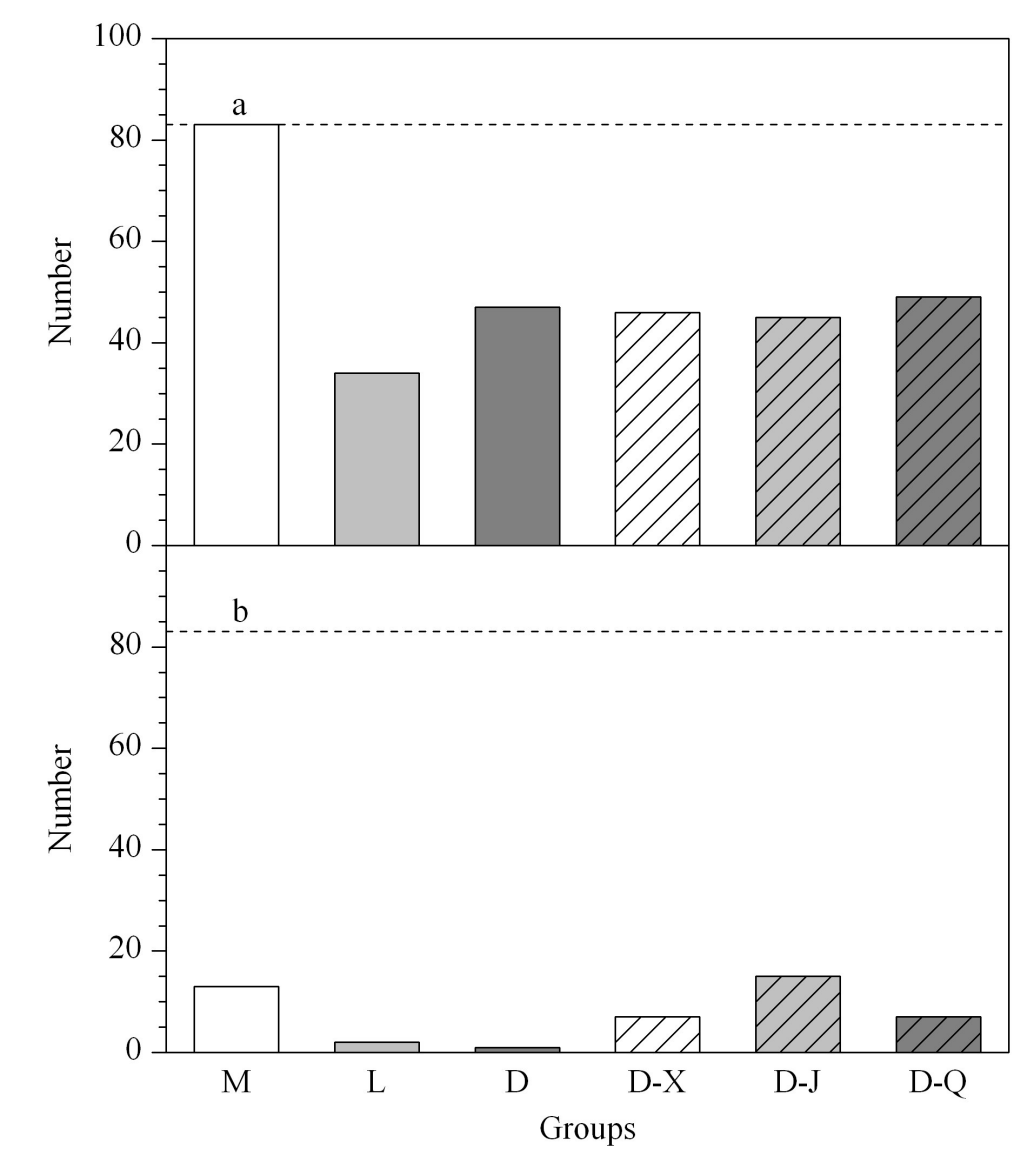
(a)第3天和(b)第7天时各组中与RSV感染有相同变化趋势的显著性代谢物的个数

根据配对*t*检验的结果,第3天时利巴韦林组或定喘汤组有药效的代谢物总共为56个。为了阐述药物治疗改变的代谢物的种类及规律,将第3天这56个代谢物校正到相对应天数正常组的相对强度显示于图7。图7中“1”表示在RSV感染后的模型组的第3天相对第0天显著性累积的代谢物;“2”表示在RSV感染后的模型组的第3天相对第0天显著性下调的代谢物;“1-a”和“2-a”表示在定喘汤组和利巴韦林组的第3天相对第0天均无显著性差异的代谢物,提示这两种药物均对这部分因RSV感染而异常的代谢物具有修正效果。“1-b”和“2-b”表示仅利巴韦林能修正的异常代谢物,它们在利巴韦林组的第3天相对第0天无显著性差异,而定喘汤对这些代谢物无明显调节作用。“2-c”表示仅定喘汤能修正的异常代谢物,它们在定喘汤组的第3天相对第0天无显著性差异,而利巴韦林无明显调节作用。“2-d”表示定喘汤和利巴韦林均可调节的代谢物,它们在感染RSV后的第3天显著性下调,在利巴韦林组的第3天相对第0天无显著性差异,但在经定喘汤干预后第3天反而显著性累积。

**图7:**
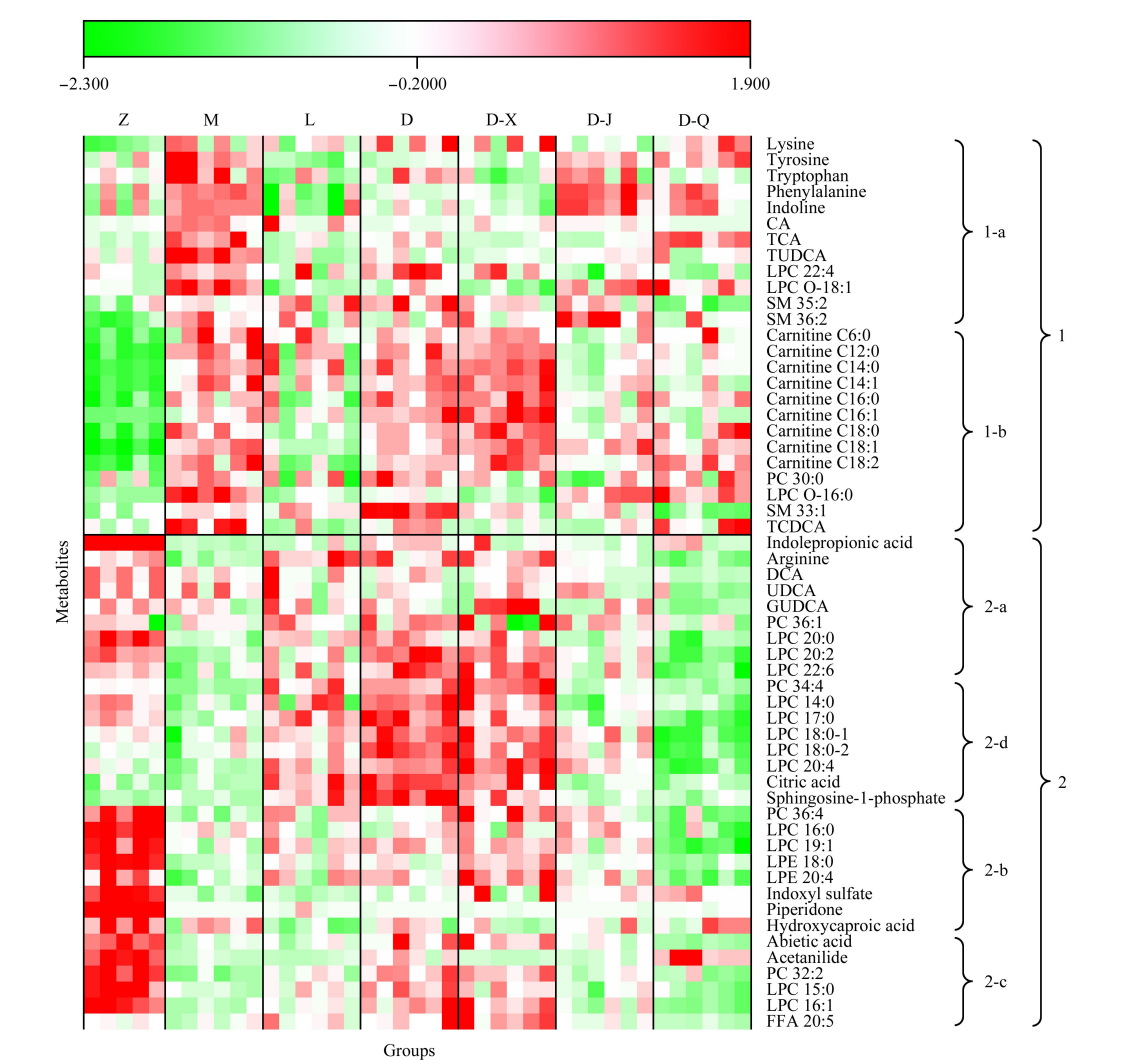
与RSV感染相关的56个代谢物在各组第3天含量的热图

如图7的“1”部分所示,与正常组相比,模型组中氨基酸(赖氨酸(lysine)、酪氨酸(tyrosine)、色氨酸(tryptophan)和苯丙氨酸(phenylalanine))、部分初级胆汁酸(胆酸(CA)、牛黄胆酸(TCA)和牛磺鹅脱氧胆酸(TCDCA))、酰基肉碱和鞘磷脂等多种代谢物的含量上调。在图7的“2”部分中,与正常组相比,模型组中精氨酸、部分次级胆汁酸(脱氧胆酸(DCA)、熊去氧胆酸(UDCA)和甘氨熊脱氧胆酸(GUDCA))、有机酸(柠檬酸(citric acid)、羟基己酸(hydroxycaproic acid)和松香酸(abietic acid))、肠道菌群相关代谢物(硫酸吲哚酚(indoxyl sulfate)、哌啶酮(piperidone)和吲哚丙酸(indolepropionic acid))、溶血磷脂酰磷脂胆碱以及溶血磷脂酰乙醇胺(LPE)等多种代谢物下调。这些代谢物可能与RSV致病过程密切相关。

氨基酸如苯丙氨酸、色氨酸、酪氨酸和赖氨酸等在感染RSV后的第3天显著性积累,而精氨酸显著性降低(如图7的“a”部分所示)。氨基酸异常提示RSV感染造成三羧酸循环,糖酵解和尿素循环等多个代谢通路改变^[26]^。这些氨基酸在定喘汤组和利巴韦林组,与模型组相比,其含量与正常组含量更接近。它们在定喘汤组和利巴韦林组内第3天和第0天间均无显著性差异,说明这两种药物均能有效修复RSV感染引起的多条氨基酸相关通路异常。此外,图7也显示了代谢物在给予3种中药分解剂干预后的含量变化。3种分解剂中,宣法组与定喘汤组相同,氨基酸含量与正常组接近,在组内第3天和第0天间均无显著性差异;降法组中各氨基酸的含量与模型组接近,在组内第3天和第0天间均存在显著性差异;清法组中部分氨基酸含量与正常组接近(如图7的“a”部分)。通过该结果推测定喘汤对氨基酸的修正药效主要来自于宣法分解剂,且3种分解剂中降法分解剂对氨基酸的修正效果最弱。

氨基酸也可在肠道中经由肠道菌群分解代谢,如色氨酸通过肠道生孢梭菌分解产生吲哚丙酸进入宿主血液^[27]^。吲哚丙酸在模型组中显著性降低,说明感染RSV后大鼠的肠道菌群代谢发生改变,先天免疫系统和适应性免疫系统发生紊乱^[27]^。吲哚丙酸在利巴韦林组、定喘汤组及3种分解剂的第3天和第0天间均无显著性差异,说明各药物均有修正肠道菌群和免疫系统异常的药效。

胆汁酸也是和肠道菌群相关的代谢物,它们能在肠道和肝脏之间进行肝肠循环^[28]^。我们之前的研究已经发现RSV感染会引起胆汁酸中CA、TCA和牛磺熊去氧胆酸(TUDCA)的异常累积,而定喘汤可有效修正这些胆汁酸水平的异常^[19]^。本研究进一步发现次级胆汁酸中的DCA、UDCA和GUDCA在RSV感染后的模型组异常下调(如图7的“2-a”部分所示),且这6种胆汁酸在定喘汤组和利巴韦林组的第3天和第0天间均无显著性差异。从热图可以看到,与模型组相比,它们在定喘汤组和利巴韦林组的第3天的含量均更接近正常组,说明这两种药物均有助于维持肠道代谢和肝肠循环的稳定。此外如图7所示,大鼠服用3种中药分解剂后,胆汁酸的含量相比模型组更接近正常组,且这6种胆汁酸在宣法组和降法组的第3天和第0天间均无显著性差异(*p*>0.05)。

至于脂类代谢物,相对正常组来说,SM的含量在模型组中显著性累积,而LPE、LPC和PC的含量显著性下调(如图7所示)。研究表明脂质水平与炎症反应密切相关^[29]^。感染病毒后,机体可通过炎症反应来抵抗病毒入侵,帮助组织修复等^[30]^。给予定喘汤和利巴韦林可修正部分脂类异常,使其含量更接近正常组(如图7所示),提示两种药物可能通过抵御病毒,降低了机体自身炎症反应。此外,如图7的“2-d”所示,对于在模型组中下调的一部分LPC和PC,利巴韦林和定喘汤均同时具有调节效果。但这些代谢物仅在经利巴韦林干预后在第3天和第0天间无显著性差异,在经定喘汤干预后反而显著性累积。如图7所示,在3种分解剂中,定喘汤组和宣法组的脂质变化趋势更为接近,说明定喘汤对脂质代谢物的影响主要来源于宣法分解剂。值得一提的是,如图7的“2”部分所示,清法组中脂类代谢物的含量明显低于其他各组,推测清法分解剂有降低大鼠血脂的功效。

酰基肉碱(尤其是中长链肉碱)的含量在模型组相对正常组累积,而在利巴韦林组中的含量更接近正常组,且利巴韦林组在第3天和第0天间无显著性差异(如图7“1-b”部分所示)。酰基肉碱与机体能量代谢密切相关^[31,32]^,而且有研究人员发现长链酰基肉碱累积可能导致肺功能损伤^[33]^。我们推测利巴韦林或可通过调节酰基肉碱来维持能量代谢的稳定,并减少肺损伤。对于各中药组,虽然酰基肉碱在定喘汤组和宣法组中的含量与模型组相似,但降法组和清法组中酰基肉碱的含量相对模型组明显下调,接近正常组(见图7“1-b”部分)。

此外,如图7的“2-c”部分所示,有机酸(羟基己酸、松香酸)在模型组中与正常组相比显著性下调,在定喘汤组中的含量与正常组接近,而在利巴韦林组与模型组接近。该结果提示与利巴韦林组相比,定喘汤有更强的维持有机酸类代谢物稳定的效果。

## 3 结论

本实验基于液相色谱-质谱联用技术研究了大鼠感染RSV后的代谢异常,考察了中药定喘汤对RSV感染大鼠代谢异常的调控作用,并比较了定喘汤的3种分解剂的药效。结果表明RSV感染可造成大鼠胆汁酸、氨基酸、酰基肉碱、脂类等多种代谢物发生紊乱。定喘汤能修正RSV引起的胆汁酸、氨基酸、有机酸水平异常,肠道菌群和免疫系统紊乱等,对于脂类代谢物的异常也有一定的改善效果。在3种中药分解剂中,定喘汤与宣法分解剂对代谢组的调控作用更为接近,可以推测宣法分解剂的组成成分麻黄和白果可能为定喘汤的主要药效来源。本研究虽然能为RSV感染的临床治疗提供一定的指导依据,但药物的作用机制仍然需要更进一步的研究。
